# Is Hepatitis C Associated with Atherosclerotic Burden? A Systematic Review and Meta-Analysis

**DOI:** 10.1371/journal.pone.0106376

**Published:** 2014-09-03

**Authors:** He Huang, Rongyan Kang, Zhendong Zhao

**Affiliations:** MOH Key Laboratory of Systems Biology of Pathogens, Institute of Pathogen Biology, Chinese Academy of Medical Sciences & Peking Union Medical College, Beijing, China; Centers for Disease Control and Prevention, United States of America

## Abstract

**Background and Aims:**

Increasing evidence demonstrates that hepatitis C virus (HCV) infection is associated with atherosclerosis. However, there are contrasting findings in several studies that the atherosclerotic burden is not associated with HCV infections. Therefore, we performed a meta-analysis to clarify if HCV infection is associated with atherosclerosis compared to non-infected people.

**Methods:**

Standard guidelines for performance of meta-analysis were followed.

**Results:**

A thorough database search performed by two independent investigators identified 14 eligible studies for analysis. The data from 11 studies were synthesized to report unadjusted odds ratios (ORs) for carotid atherosclerosis; the pooled unadjusted OR (95% confidence interval (CI)) was 1.65 (1.21, 2.09). By synthesizing the data from 8 studies to report adjusted ORs for carotid atherosclerosis the pooled multi-confounder adjusted OR (95% CI) was 1.76 (1.20, 2.32). However, the numbers of studies on coronary or femoral atherosclerosis were limited and not enough for analysis.

**Conclusions:**

Our meta-analysis indicats that HCV infection is associated with carotid atherosclerosis independent of classical risk factors. Therefore, we would recommend for HCV infected patients to be counseled on their risk for carotid atherosclerosis.

## Introduction

Hepatitis C virus (HCV) infection is a global health problem, with an estimated global prevalence of 3%, resulting in more than 170 million infected people [1].

Despite of inducing intra-hepatic diseases such as liver cirrhosis and hepatocellular carcinoma, HCV infection might also accelerate the disease process of diabetes [2], [3], various other malignances [4], [5], and genitourinary conditions [6], [7]. A positive association between hepatitis C and cardiovascular disease (CVD) has also been found in the last decade [8]–[13]. As a major cause of CVD, atherosclerosis was found to be accelerated in HCV infected patients in several studies [14]–[23]. However, other reports did not find an excess significant risk [24]–[28]. Further, Bilora et al. found that HCV infection delayed the process of atherosclerosis [29]. Differences in study design, patient source, case definition, and sample size may explain the observed variability discovered among studies.

Until present, there were four systematic reviews published on the association between HCV and CVD [30]–[33]. Aslam et al. performed a systematic review to study the association between carotid atherosclerotic plaques and HCV infection; however, a standard meta-analysis was not performed and several new studies have been published after that. [30] In Aslam's study, the percentage of subjects with carotid plaques was not significantly different between HCV+ and HCV- subjects (*P* = 0.05) and the results were not adjusted for other risk cofounders. Roed et al. performed a meta-analysis on the association between HCV and coronary artery diseases (including coronary atherosclerosis), but they did not perform subgroup analysis on coronary atherosclerosis [31]. In Adinolfi's review, the authors systematically reviewed studies on the association between HCV and atherosclerosis. Given the conflicting results in the field, the authors concluded that meta-analyses are needed to reveal the association between HCV infection and atherosclerosis [32]. Very recently, Wong et al. performed a systematic review on HCV and coronary artery disease risk, but they did not perform a meta-analysis to reveal the relevance between HCV infection and coronary atherosclerosis. [33] The purpose of this study was, therefore, to conduct meta-analyses to assess whether HCV infection is associated with carotid, coronary, or femoral atherosclerosis compared to normal subjects. In addition to providing a greater understanding about the association between chronic hepatitis C infection and atherosclerotic burden, the findings presented herein may also help inform clinical practice guidelines and suggest gaps in the current understanding that may be important to address in future studies.

## Methods

This systematic review and meta-analysis was performed following the guidance provided in the Cochrane Handbook [34] and is reported according to the Meta-analysis of Observational Studies in Epidemiology (MOOSE) guidelines [35].

### Information Sources and Search Strategy

To identify all potential eligible studies, two investigators independently searched databases including Medline, Embase, Conference Proceedings Citation Index-Science (CPCI-S), and Cochrane library. The searches included combination from a list of key words indicated in Table S1. The detailed search strategy and results are also shown in Table S1. Searches were current as of February 10, 2014. The citations of eligible studies and relevant review articles were reviewed to identify additional studies not captured by our database searches. Google scholar was also used to identify additional studies.

### Eligibility Criteria and Study Selection

All published epidemiologic studies providing, or with data to calculate, an estimate of atherosclerotic burden in hepatitis C infected (HCV+) adults compared to adults without infection (HCV-) or an estimate of the burden of HCV infection in adults with atherosclerosis compared to adults without atherosclerosis were considered for possible inclusion in the current meta-analysis. HCV infection was diagnosed by anti-HCV antibody ELISA, HCV protein ELISA, or HCV RNA PCR. The method of artery assessment was limited to arteriography or B-mode ultrasound; atherosclerosis should be defined according to the measurement of intima media thickness (IMT) or the observation of stenosis. The minimum number of the total sample size in an eligible study was limited to 40.

Studies were excluded if: (1) the exposed and unexposed groups came from different geographical and temporally defined underlying populations; (2) the studies included children, post-transplant recipients, or pregnant women; (3) the studies did not provide risk estimates or data necessary to calculate them; or (4) the studies were not published in English. If different overlapping studies used same study population, only the largest eligible report was included. If a study was updated to increase the sample size, the updated study was included. If a study was updated with more sensitive diagnostic methods, the updated study w included, because the updated study might increase the accuracy of the results.

Two investigators independently reviewed the titles, abstracts, and manuscripts of the captured studies to determine if an individual study was eligible for meta-analysis. If disagreements appeared, a third investigator helped to resolve the problem.

### Data Collection

Data on study methods and results were independently extracted by two investigators. The following data were extracted from each study: the first author's last name, publication year, country or region where the study was conducted, study design, number of subjects, HCV positivity criteria, method of artery assessment, definition of atherosclerosis, unadjusted and multi-adjusted estimates with corresponding 95% confidence intervals (95% CI), matching variables, and cofounders were adjusted for in the multivariate analysis. If original data were provided, unadjusted risk estimates were calculated by us.

### Analysis

The summary odds ratio (OR) and the corresponding 95% CI was calculated using random-effects models of DerSimonian and Laird, [36], [37] which incorporate both within- and between-study variability's, as a weighted average of the estimated ORs, by giving each study a weight proportional to its precision. All meta-analyses were presented as forest plots with estimates for all individual studies as well as the overall pooled estimator. Shaded figures provided for all individual study estimates had dimensions proportional to their weight in calculation of the pooled estimator.

Statistical heterogeneity among studies was evaluated using the *I* ^2^ of Higgins and Thompson, which quantifies the proportion of total variation attributable to between-study differences or heterogeneity as opposed to random error or chance [38]. An *I* ^2^>50% or *P*<0.10 was employed to determine if significant between-study heterogeneity existed [38]. Publication bias was assessed with Begg's and Egger's tests [38] and the Begg's funnel plot was presented in this study [39]. Sensitive analysis by excluding each study in the meta-analysis was performed to assess whether any one study had a dominant effect on the pooled estimates and to determine the resources of heterogeneity. A subgroup analysis was also performed according to study characteristics.

All analyses were conducted using STATA 12.0 with user-written meta-analysis commands (StataCorp LP, College Station, TX, USA).

## Results

### Searches

**Figure 1:**
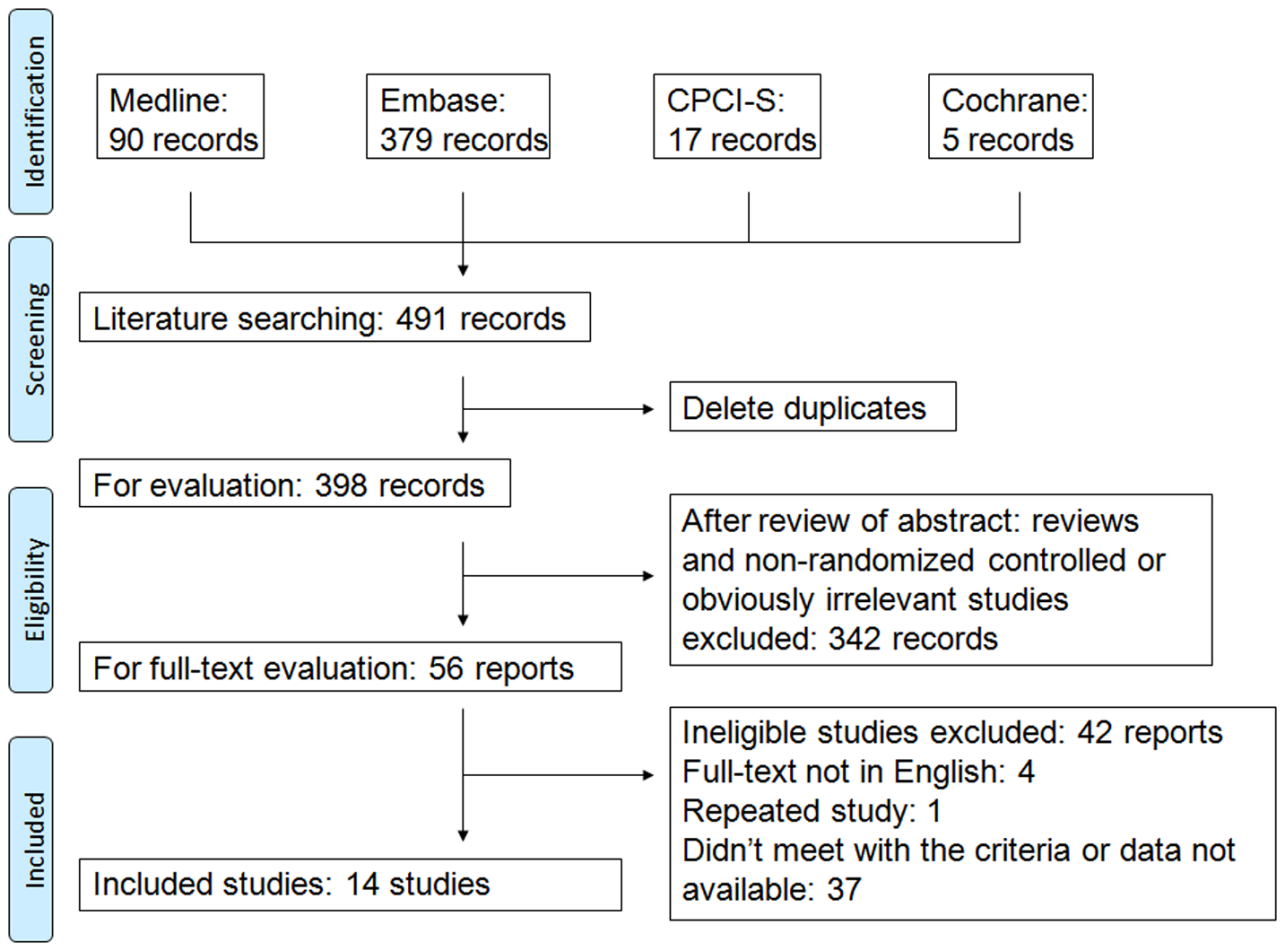
Flow diagram of study identification.

Figure 1 shows the selection of eligible studies. A total of 491 records were retrieved from the four databases indicated above. After deletion of duplicate studies, 398 potentially eligible records were identified. A review of abstracts resulted in the exclusion of 342 reports. Full-text evaluation of the 56 left resulted in the inclusion of 14 reports in this analysis.

### Study Characteristics

The main characteristics of the 14 identified reports are showed in Table S2, including 11 studies on carotid [14], [16]–[21], [25], [26], [28], [40], 2 studies on coronary [15], [24], and 1 study on carotid and femoral [41]. Unadjusted ORs were collected or calculated from the 14 articles and adjusted ORs could be collected from 10 articles.

### Unadjusted OR of Carotid Atherosclerosis

**Figure 2:**
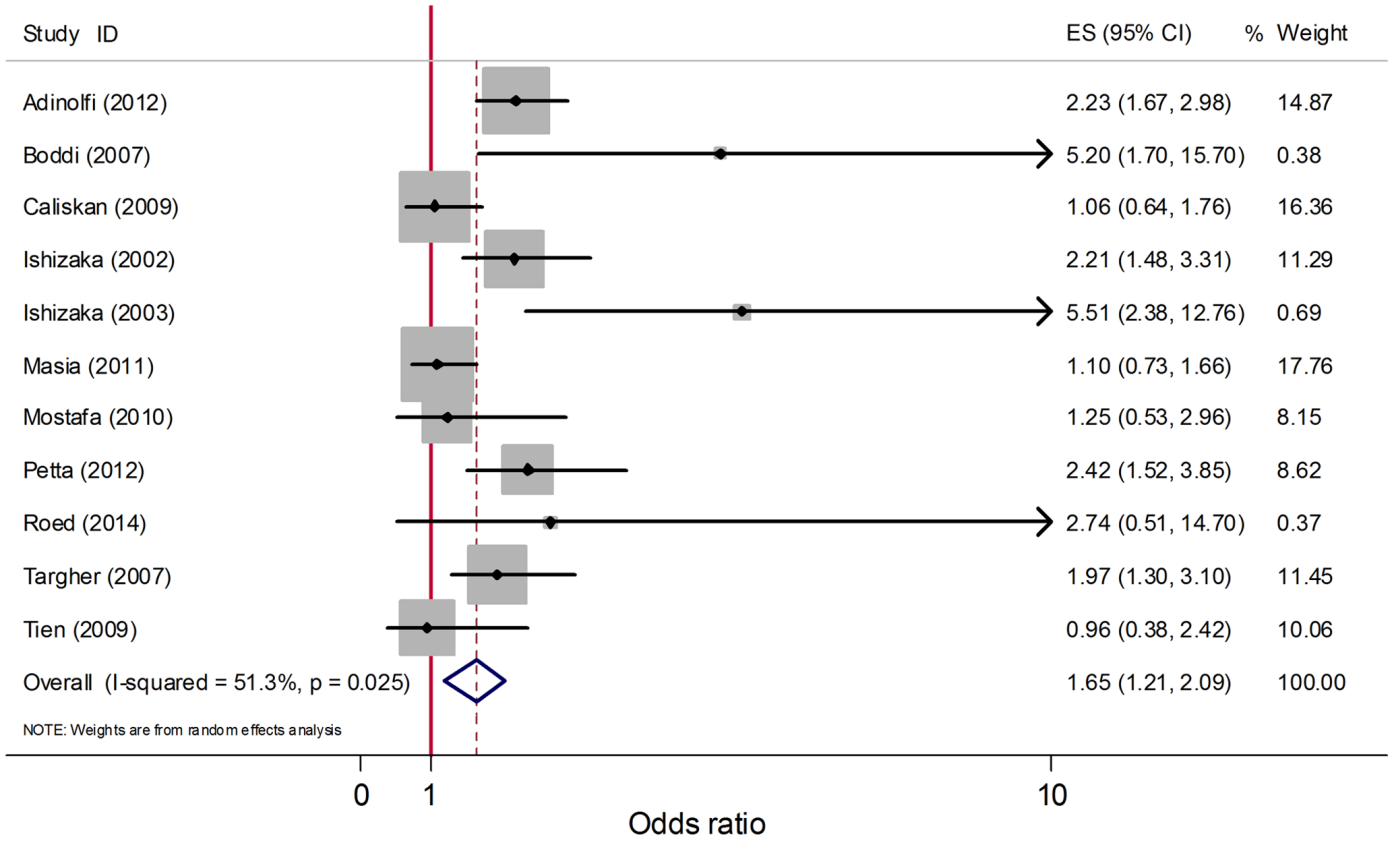
Forest plot for meta-analysis comparing unadjusted OR of carotid atherosclerosis in HCV infected patients compared to that in non-infected controls. Unadjusted ORs from included studies and the pooled OR are shown. Dimension of shaded OR for individual studies is proportional to their total weight in calculation of the pooled estimator.

All of the 11 studies on carotid atherosclerosis either reported univariate estimates of carotid atherosclerosis with HCV or provided data for calculating it. By synthesizing these 11 studies, the pooled unadjusted OR (95% CI) was 1.65 (1.21, 2.09) (Figure 2). *I* ^2^ = 51.3% and *P* = 0.025 suggest that heterogeneity existed. Publication bias was not evident as calculated by Egger's test (*P* = 0.938) and Begg's funnel plots (Figure S1). Sensitive analysis did not find any studies that had a dominant effect on the results (Figure S2). However, a sensitive analysis showed that the study by Masia in 2011 and the study by Caliskan in 2009 were the major sources of heterogeneity and affected the results most significantly. After removing these two studies, the pooled OR (95% CI) was 1.95 (1.53, 2.38) (*I* ^2^ = 13.3%, *P* = 0.323).

### Adjusted OR of Carotid Atherosclerosis

**Figure 3:**
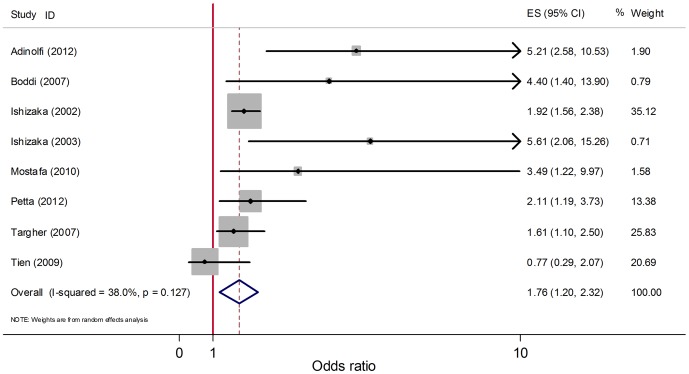
Forest plot for meta-analysis comparing adjusted OR of carotid atherosclerosis in HCV infected patients compared to that in non-infected controls. Eight studies reporting unadjusted ORs were included. Unadjusted ORs from the included studies and the pooled OR are shown. Dimension of shaded OR for individual studies is proportional to their total weight in calculation of the pooled estimator.

In most of the studies included, the control groups were only matched for age and gender. Due to other factors, including smoking, hypertension, and diabetes, there may be an increased risk of atherosclerosis; therefore, the multi-cofounder adjusted estimates should be analyzed to determine if HCV infection was associated with atherosclerosis independently. By integrating 8 studies reporting multivariate ORs for carotid atherosclerosis, the pooled adjusted OR (95% CI) was 1.76 (1.20, 2.32) (Figure 3). An *I* ^2^ = 38.0% and a *P* = 0.127 did not suggest that there was heterogeneity. Publication bias was not suggested when calculated by Egger's test (*P* = 0.261) and Begg's funnel plots (Figure S3).

### Subgroup Analysis of Adjusted Risk of Carotid Atherosclerosis

To minimize the risk of bias caused by hospital-based studies, we performed a subgroup analysis of studies in which community-based controls were used. The increased risk of carotid atherosclerosis was also observed in this subgroup. The pooled adjusted OR (95% CI) was 1.92 (1.37, 2.46) (Table 1). However, the summary of hospital-based controlled studies did not suggest an association (OR: 1.47; 95% CI: 0.22, 2.72).

**Table 1:** Subgroup analysis of the adjusted risk of carotid atherosclerosis with HCV infection.

Subgroup	Categories	Studies, n	Adjusted OR (95% CI)
Selection of controls	Community-based	5	1.92 (1.37, 2.46)
	Hospital-based	3	1.47 (0.22, 2.72)
HCV positivity criteria	PCR	4	2.00 (1.13, 2.88)
	Anti-HCV ELISA	2	1.93 (1.52, 2.34)
Method of artery assessment	Single investigator	3	1.94 (1.54, 2.35)
	Two investigators	3	1.76 (1.15, 2.37)
Location	Italy	4	2.01 (1.10, 2.93)
	Other places	4	1.74 (1.37, 2.11)
Sample size	each group >100	4	2.01 (1.54, 2.47)
High quality studies only*		4	1.91 (1.29, 2.52)
Removing the study on female population		7	1.91 (1.57, 2.25)

* As estimates in multivariate analyses were fully-adjusted, "Comparability" was not considered in Newcastle-Ottawa Scale in this study (maximum score = 7). Score = 7 was defined as high quality.

As the HCV positive criteria reflected the status of infection and might affect the results, we performed subgroup analyses of studies utilizing HCV RNA PCR or anti-HCV ELISA. As shown in Table 1, both studies suggested a positive association between HCV and carotid atherosclerosis.

All of the studies utilized ultrasound to diagnose atherosclerosis and could be divided to two groups according the number of participating investigators (one single investigator or two investigators). As show in Table 1, both of the subgroup analyses suggest a positive association.

Of the 8 studies included in the analysis of adjusted ORs of carotid atherosclerosis, 4 studies were performed in Italy [14], [16], [20], [21]. The pooled OR (95% CI) of these studies was 2.01 (1.10, 2.93) (Table 1), suggesting that the association between carotid atherosclerotic burden and HCV infection was positive in the Italian population. Comparing other study populations also suggested a positive association (Table 1).

Subgroup analysis in which only high quality studies (according to the Newcastle-Ottawa Scale, Table S3) were included, studies with sample sizes less than 100 were excluded, or the study only on females was excluded [25] also showed a positive association (Table 1).

### The Association between Coronary or Femoral Atherosclerosis and HCV

The database search retrieved two studies on coronary [15], [24] and one study on femoral [41]. The number of eligible studies in these subgroups was not enough for a meta-analysis.

## Discussion

To our knowledge, this was the first meta-analysis performed to study the association between atherosclerotic burden and HCV infection. Our meta-analysis which combined the multi-cofounder adjusted odds ratios from eligible studies demonstrated a significant increase in carotid atherosclerotic burden with HCV infection.

A recent published meta-analysis by our group showed a positive association between HCV infection and stroke. [42] As an important risk factor of stroke, carotid atherosclerosis in HCV infected patients was worth researching. We performed an exhaustive search to identify all eligible studies. To improve the quality of included studies, we pre-specified eligibility criteria including a minimum total sample size. After identification of eligible studies, the unadjusted and adjusted estimates for carotid atherosclerotic burden with HCV infection could be calculated. The final meta-analysis of unadjusted OR for carotid atherosclerosis suggested a positive association between HCV and carotid atherosclerosis. However, significant heterogeneity was observed. A sensitive analysis demonstrated that the study by Caliskan (2009) and the study by Masia (2011) were the major source of heterogeneity and had the most significant affect on the final results. Compared to the other included studies, Caliskan (2009) and Masia (2011) might induce heterogeneity for the following reasons: 1) these studies defined a plaque as IMT≥1.0 mm, but the value in the other studies was a minimum of 1.2 mm; and 2) exposed and control groups were not matched for any variables.

The final meta-analysis of adjusted OR for carotid atherosclerosis included 960 exposed individuals and 8122 control subjects from 8 studies, which may allow for a greater possibility of reachingstrong conclusions. Our conclusion was based on the analysis of studies providing cofounder-adjusted ORs; we believe that our statistical methodology provides robust results. Among the 8 studies, 7 studies reported a positive association and only Tien's study reported a negative association [25]. Thus, it was not surprising that the meta-analysis demonstrated a positive association between carotid atherosclerosis and HCV. The study by Tien et al. was performed only in a HIV-infected female population, which might explain the variance. The subgroup analysis of studies performed in hospitals resulted in conflicting results possibly due to the smaller sample size and complicated subject characteristics. The subgroup analyses of studies conducted in an Italian population, and the other populations, suggests that the results from Italian population can be extrapolated to populations. Other subgroup analyses based on HCV positive criteria and methods of artery assessment, also showed a positive association, suggesting that these factors did not have an impact on the results.

The underlying mechanism of the association between carotid atherosclerotic burden and HCV infection has not been fully-delineated. The literature provides some possible mechanisms to explain this association. Evidence has shown that serum HCV RNA levels are independently associated with carotid atherosclerosis [14] and HCV RNA was isolated inside of plaques, suggesting that HCV replicates within plaques [16], [43]. Due to damage caused directly to arteries, HCV might stimulate the synthesis of pro-inflammatory cytokines to exert a pro-atherogenic activity [44], [45].

In evaluating the findings from our meta-analysis, some limitations should be noted. First, our restriction to studies published in English might have resulted in exclusion of studies of particular ethnic groups. However, only 2 studies evaluated were not published in English, even though most of the included studies were conducted in countries where English is not the primary language. Second, the HCV infection positive criterion was different in the included studies. In most studies analyzed, acute infection, persistent chronic infection, and cured infection were not specified. Although publication bias was not found, the outcomes of these situations might be different. Third, the sizes of plaques for definition of atherosclerosis were different among the studies, which may induce bias. However, publication bias was not found in our analyses. Fourth, only one study was prospective [20] and the others were retrospective. The inherent limitations of retrospective studies might have influenced our findings. More studies with prospective design will be needed. Fifth, only one study specified the genotype of HCV, and they found that HCV genotype 1 significantly increased the risk of carotid atherosclerosis [20]. More studies on atherosclerosis risk with different genotypes of HCV will provide more in depth information. Due to the limitations mentioned above, the results of this meta-analysis should be interpreted with care.

In conclusion, our meta-analysis revealed that HCV infection is significantly associated with carotid atherosclerotic burden. In addition, we speculate that HCV infection might increase the risk of carotid atherosclerosis. Due to the limitations mentioned above, more population-based well-designed cohort studies are be needed to verify if HCV infection accelerates the progression of atherosclerosis. More studies are also needed to evaluate the risk of coronary and femoral atherosclerosis with HCV infection. Furthermore, future studies may evaluate the impact of different genotypes of HCV infection on atherosclerosis. The updating of this meta-analysis will give us more information and may help inform clinical practice guidelines in the future.

## Supporting Information

Figure S1 **Begg's funnel plot (with pseudo 95%CIs) for the meta-analysis of unadjusted OR for carotid atherosclerosis with HCV infection to detect any publication bias.**
(TIF)

Figure S2 **Sensitive analysis in meta-analysis of unadjusted OR for carotid atherosclerosis with HCV infection.**
(TIF)

Figure S3 **Begg's funnel plot (with pseudo 95% CIs) for the meta-analysis of adjusted OR for carotid atherosclerosis with HCV infection to detect any publication bias.**
(TIF)

Table S1 **Search Strategies and Results.**
(XLS)

Table S2 **Characteristics of identified studies examing association between atherosclerosis and HCV infection.**
(XLS)

Table S3 **Quality assessment of individual studies using Newcastle-Ottawa Scale for case-control studies.**
(XLS)

Checklist S1 **PRISMA Checklist.**
(DOC)
