# Supplementary figures and images for: Is Hepatitis C Associated with Atherosclerotic Burden? A Systematic Review and Meta-Analysis
Source: PLoS One. 2014 Sep 3;9(9):e106376. doi: 10.1371/journal.pone.0106376 (PMC4153605; doi:10.1371/journal.pone.0106376)

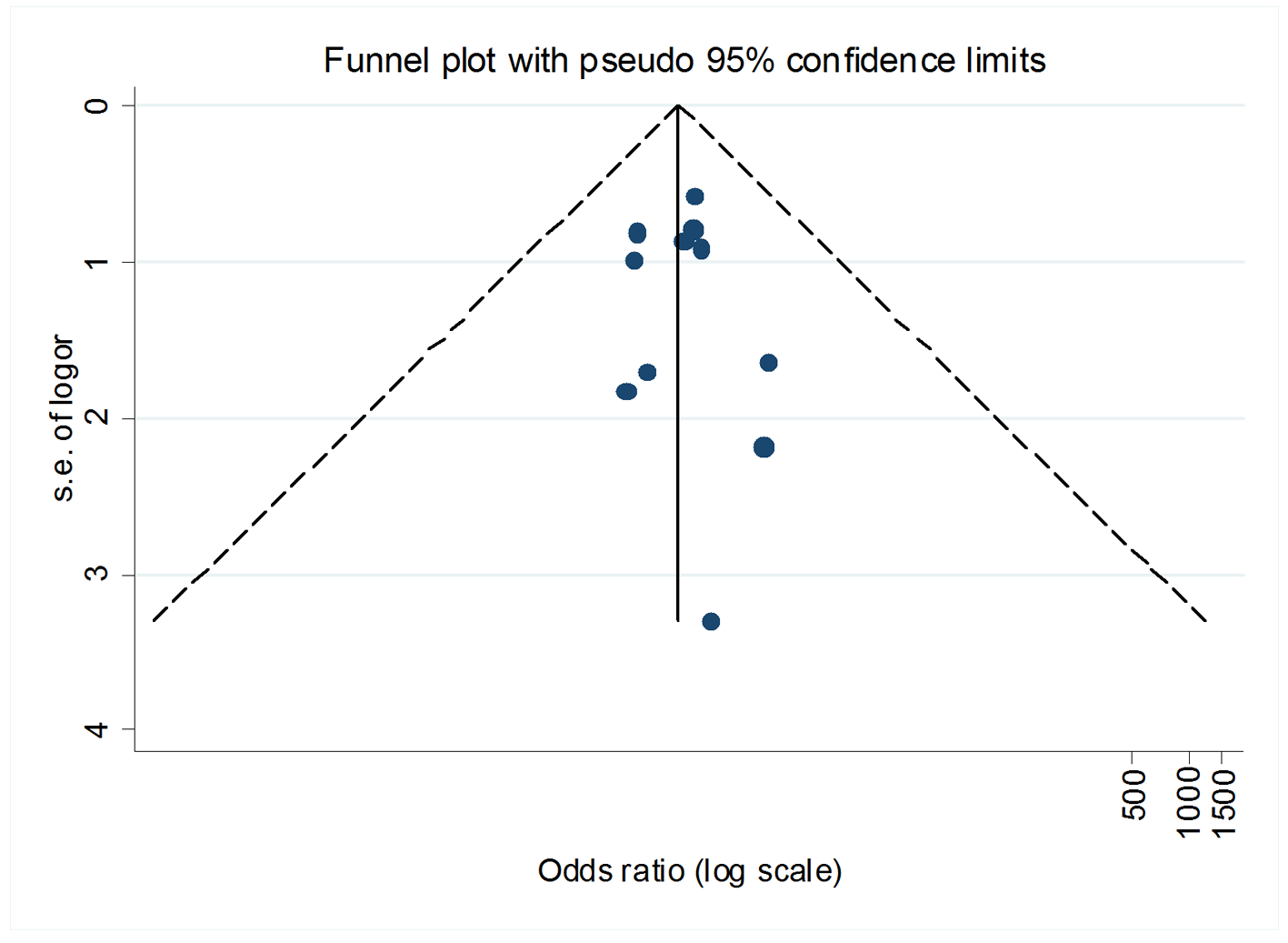

Supplement: Figure S1 — Begg's funnel plot (with pseudo 95%CIs) for the meta-analysis of unadjusted OR for carotid atherosclerosis with HCV infection to detect any publication bias. (TIF) [file pone.0106376.s001.tif]

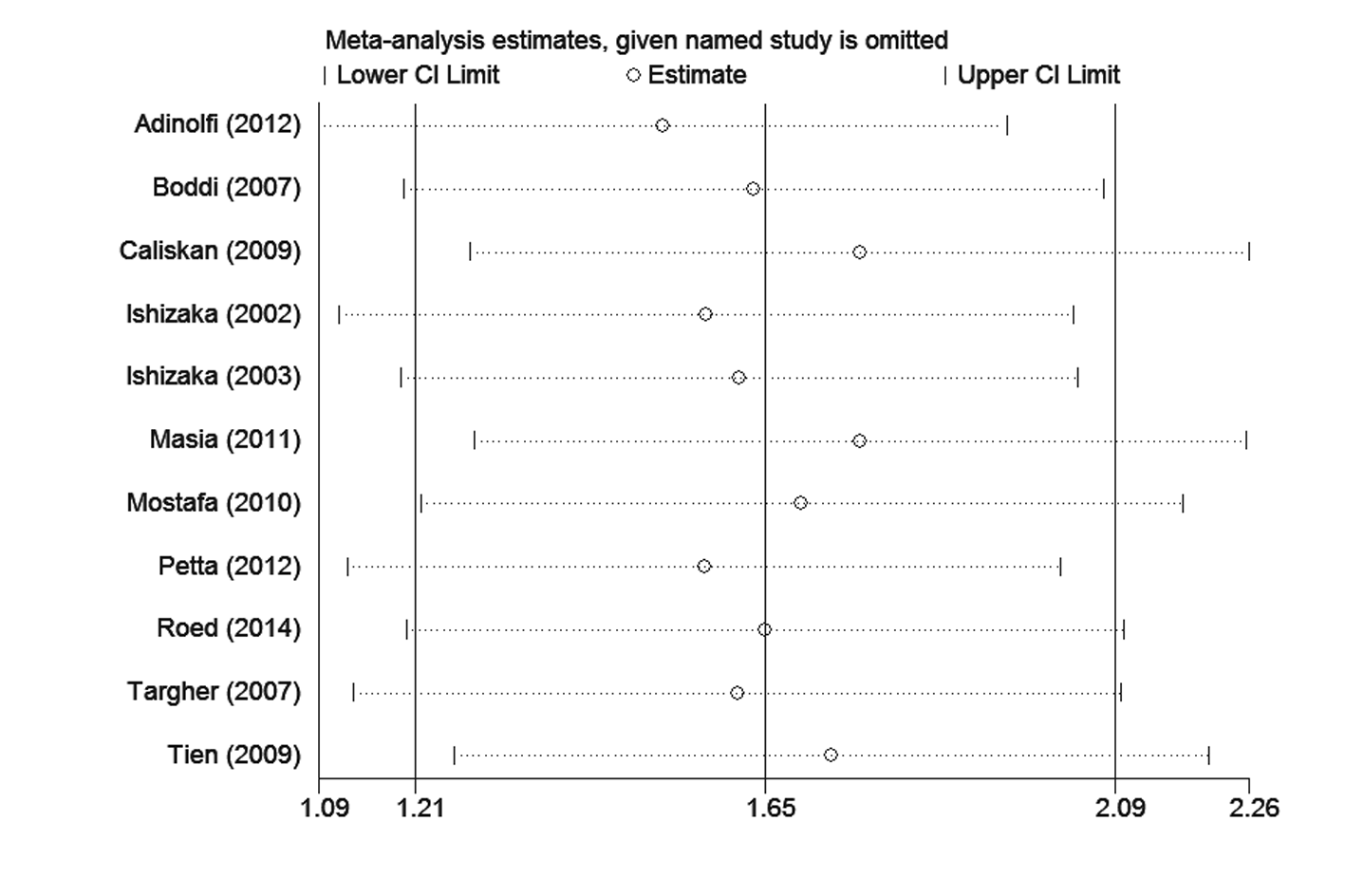

Supplement: Figure S2 — Sensitive analysis in meta-analysis of unadjusted OR for carotid atherosclerosis with HCV infection. (TIF) [file pone.0106376.s002.tif]

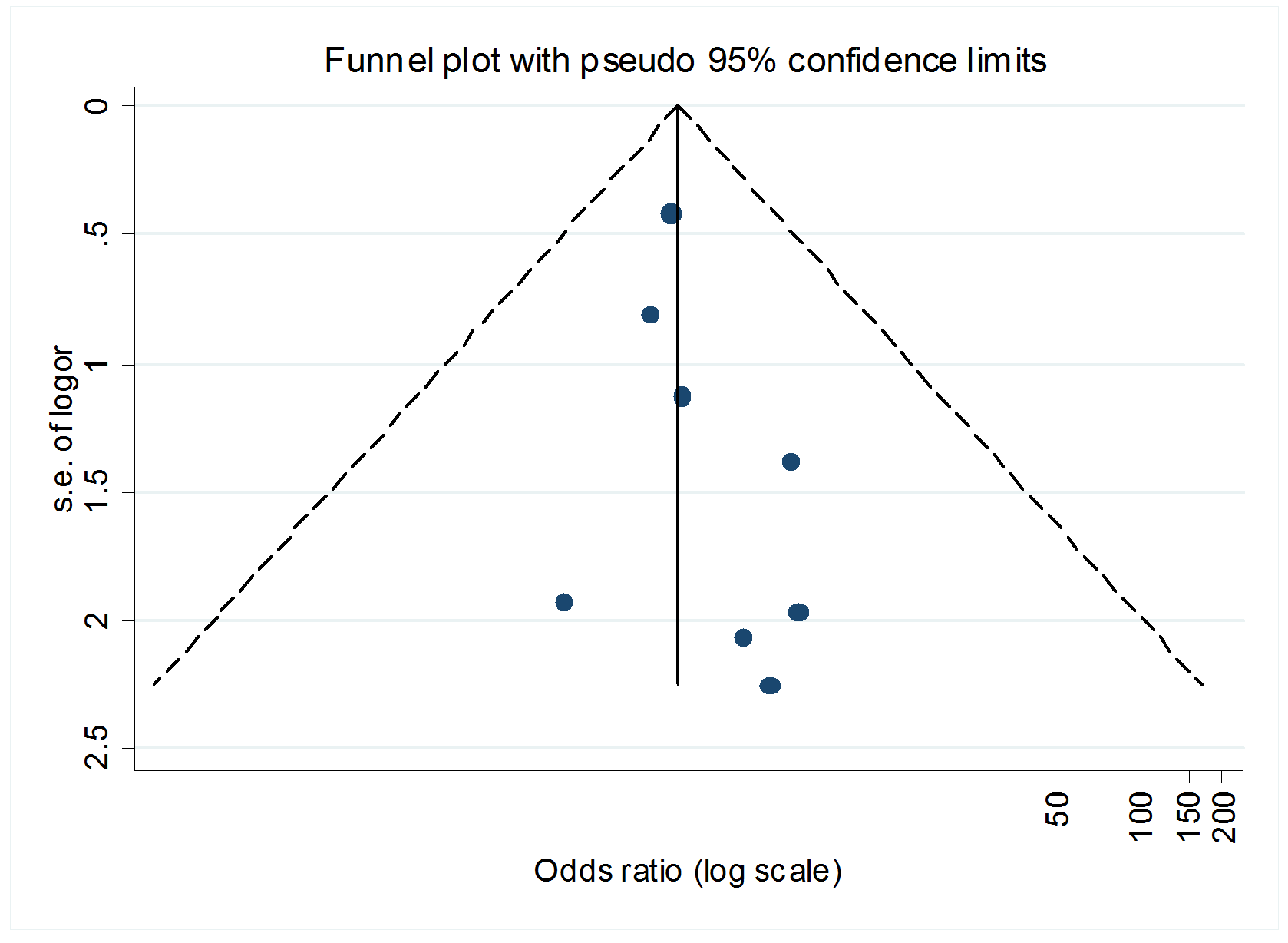

Supplement: Figure S3 — Begg's funnel plot (with pseudo 95% CIs) for the meta-analysis of adjusted OR for carotid atherosclerosis with HCV infection to detect any publication bias. (TIF) [file pone.0106376.s003.tif]
